# Corresponding Active Orbital Spaces along Chemical Reaction Paths

**DOI:** 10.1021/acs.jpclett.2c03905

**Published:** 2023-02-20

**Authors:** Moritz Bensberg, Markus Reiher

**Affiliations:** ETH Zürich, Laboratorium für Physikalische Chemie, Vladimir-Prelog-Weg 2, 8093 Zürich, Switzerland

## Abstract

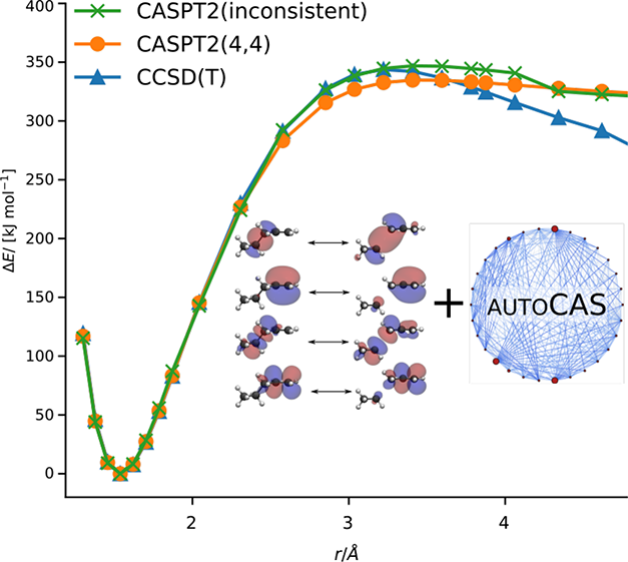

The accuracy of reaction energy profiles calculated with multiconfigurational electronic structure methods and corrected by multireference perturbation theory depends crucially on consistent active orbital spaces selected along the reaction path. However, it has been challenging to choose molecular orbitals that can be considered corresponding in different molecular structures. Here, we demonstrate how active orbital spaces can be selected consistently along reaction coordinates in a fully automatized way. The approach requires no structure interpolation between reactants and products. Instead, it emerges from a synergy of the Direct Orbital Selection orbital mapping ansatz combined with our fully automated active space selection algorithm autoCAS. We demonstrate our algorithm for the potential energy profile of the homolytic carbon–carbon bond dissociation and rotation around the double bond of 1-pentene in the electronic ground state. However, our algorithm also applies to electronically excited Born–Oppenheimer surfaces.

Molecules featuring large conjugated π-systems, transition metal complexes, or molecules in reactions often feature close-lying, nearly degenerate frontier orbitals. For these cases of static electron correlation, multiconfigurational quantum chemical methods provide qualitatively correct electronic wave functions; examples are the complete active space self-consistent field (CASSCF),^1−4^ full configuration interaction quantum Monte Carlo^5,6^ (FCIQMC), and the density matrix renormalization group (DMRG)^7,8^ approaches. Because of the exponential scaling of the number of many-electron basis states with the number of orbitals, these approaches demand a restriction of the size of the orbital basis by defining an active orbital space for a given molecular structure. Then, all possible electronic configurations are constructed for a basis-set expansion of the electronic wave function from these active orbitals. As a consequence, the dynamic electron correlation originating from the neglected orbitals must be captured by additional procedures, of which multireference perturbation theory^9^ is a standard choice.

For accurate reaction energies, active spaces must be selected that are consistent for multiple molecular structures along a reaction path; i.e., the active spaces must consist of orbitals that can be considered corresponding between all structures along a reaction coordinate. Otherwise, the total correlation energy is calculated inconsistently, leading to an erratic behavior of the relative energies.

Here, we propose a fully automated orbital mapping procedure that ensures consistent orbital spaces between multiple structures along a cut through the Born–Oppenheimer surface. As a byproduct, the mapping procedure identifies the valence orbitals that change significantly. Since orbitals describing bonds that are broken or formed during a reaction cannot be identified in every structure, these orbitals cannot be mapped unambiguously and must be included in the active spaces. To ensure consistent active orbital spaces, all of those valence orbitals that are varying along a reaction path are assigned to the active orbital space as soon as one of them is selected for it.

Compared to the active space selection protocol for reactions proposed in ref (10), our new approach does not follow orbitals along a reaction coordinate through interpolated structures. Hence, we avoid practical problems that can arise if orbital sets change significantly, preventing direct orbital identification between structures.

For a single molecular structure, active spaces are often selected manually based on expertise or by analyzing the orbital occupation numbers from Hartree–Fock^11−13^ or Møller–Plesset perturbation theory.^14^ A reliable and convenient alternative has been the introduction^15^ of our fully automated active space construction algorithm based on single-orbital entropies^16^ obtained from approximate but fast DMRG calculations.^10,15,17−19^ We note that, after the proposition of autoCAS in 2016, various other orbital selection approaches have been proposed^20−22^ which, however, are less general, expensive to evaluate, and/or not fully automatically applicable.

Consistent orbital spaces between structures along a reaction coordinate have already been investigated for embedding calculations.^23,24^ For many embedding approaches (e.g., projection-based embedding^25^ or multilevel correlation methods^26−30^), an orbital set is selected from the occupied and localized supersystem Hartree–Fock (HF) or Kohn–Sham orbitals. Selecting these orbitals can be automatized through the direct orbital selection (DOS) approach,^24,31^ which identifies the occupied orbitals that change significantly along a path and selects them for the embedded region. The algorithm provides a bijective map between sets of occupied orbitals that can be leveraged in multilevel coupled cluster calculations.^32,33^

To obtain consistent active orbital spaces for CASSCF, FCIQMC, and DMRG calculations of structures along a path cut through a Born–Oppenheimer hypersurface, we extend the DOS mapping procedure to include also the virtual valence orbitals. We then combine the automatized orbital mapping with the automatized active space selection algorithm implemented in autoCAS.^18,34^ Finally, we demonstrate that this ansatz leads to fully automatized active orbital selections for chemical reactions in which the multiconfigurational character changes markedly along a reaction coordinate.

For each structure *L* along a path, we consider a total orbital set . This orbital set is either the set of all occupied orbitals or of all virtual valence orbitals of *L*. We note that the algorithm initially published in ref (24) and outlined below is independent of the specific occupation of the orbitals. The orbital maps are independently constructed for occupied and virtual orbitals. The algorithm constructs a map between sets of orbitals rather than between individual orbitals because this allows us to avoid the problem of defining maps between orbitals that change significantly from structure to structure. Furthermore, the mapping criteria are chosen to be invariant under translation and rotation of the molecule since these operations do not change the electronic structure.

The idea of the algorithm is to identify all orbital sets that can be identified unambiguously in all structures along the reaction coordinate and then collect all other remaining orbitals in one set  of nonmatchable orbitals.

Each orbital  is compared to all orbitals in all orbital sets  (each of which is obtained for a structure selected along a path through configuration space) through criteria characterizing its shape and spatial localization in the molecule. We explicitly avoid criteria which are directly sensitive to the molecule’s structure given in terms of its nuclear coordinates, because we are only interested in the shapes of orbitals. The mapping criteria should only characterize an orbital as a specific bond orbital or lone pair to mirror what a visual inspection of the molecular orbitals in terms of isosurface plots would deliver.

A mapping between two orbitals ψ_*iL*_ and ψ_*jK*_ with indices *i* and *j* of the structures *L* and *K* will be defined if the following condition is met:
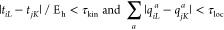
1Here, τ_kin_ and τ_loc_ are predefined thresholds that are chosen to be equal τ = τ_kin_ = τ_loc_ in practice.^24^ Furthermore, *t*_iL_ is the orbital kinetic energy of orbital ψ_*iL*_ in Hartree atomic units
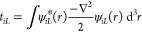
2and  is an orbital-wise population assigned to an atom or a minimal-basis-function shell *a* in the molecule. The orbital kinetic energy characterizes how compact an orbital is. In principle, the populations  can be calculated by any population analysis that provides orbital-wise populations, such as the Mulliken population analysis.^35^ We employ shell-wise intrinsic atomic orbital (IAO) populations^36^ because they are basis-set insensitive and distinguish between contributions from different shells of IAOs (e.g., 1*s*, 2*s*, 2*p*). Therefore, shell-wise IAO charges characterize on which atoms an orbital is localized as well as the orbital’s shape. The shell-wise IAO populations are given through the projection on the IAOs  (e.g., *p*_*x*_, *p*_*y*_, or *p*_*z*_ for a *p*-type IAO) of the shell *a*, with angular momentum *l*_*a*_, orientation *m*, and structure *L*.^36^
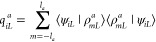
3

Evaluating the condition in eq 1 for every orbital pair  and  provides an assignment of a set of orbitals {ψ_*jK*_} to each orbital ψ_*iL*_. We collect these assignments in a map *s*_*LK*_.
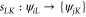
4

Based on this map, we identify the orbitals that change strongly along the reaction coordinate, i.e., between any pair of structures *LK*.

We split the total orbital set into an initially mappable orbital set  with *s*_*LK*_(ψ_*iL*_) ≠ {} *∀K* (i.e., the orbital is mappable for all *K*) and an orbital set  for which no orbital match can be identified in at least one structure *K*.

We must now ensure that the orbital mapping is consistent along the reaction coordinate. This procedure is illustrated in Figure 1. We map the orbital sets obtained from the self-map *s*_*LL*_(ψ_*iL*_) = *S*_*iL*_ between structures with the map
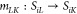
5

**Figure 1:**
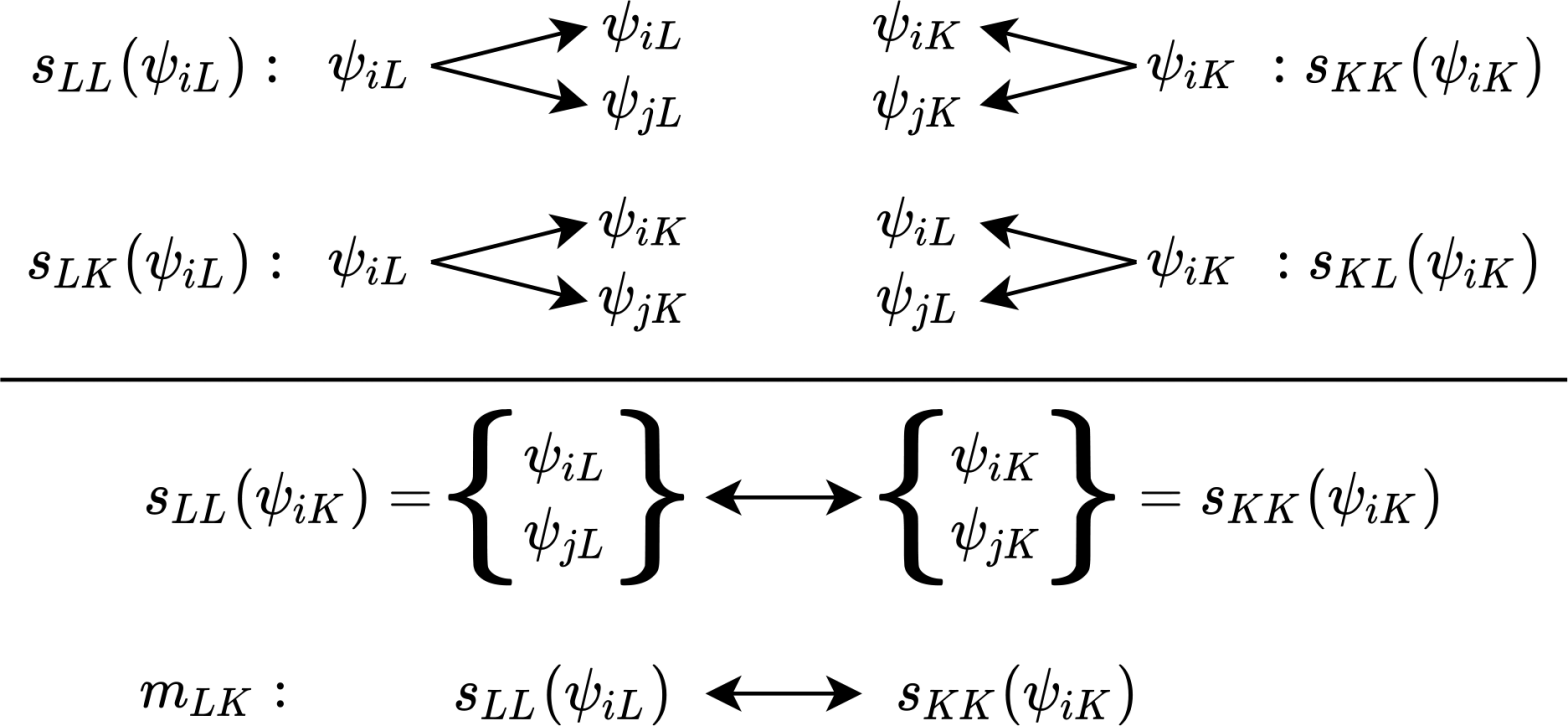
Illustration of the orbital mapping procedure. Each of the orbitals ψ_*iL*_ and ψ_*iK*_ is mapped to sets of orbitals through the maps *s*_*LK*_ and *s*_*KL*_. If these sets are the orbital sets from the self-maps *s*_*LL*_ and *s*_*KK*_, a mapping between *s*_*LL*_ and *s*_*KK*_ will be defined in *m*_*KL*_; i.e., these orbitals can be considered unchanged in structures *K* and *L*.

The condition for assigning a map from *S*_*iL*_ to *S*_*iK*_ in *m*_*LK*_ is that every orbital in *S*_*iL*_ is mapped to all orbitals in *S*_*iK*_, and not to any other orbital of *K* through the map *s*_*LK*_. Furthermore, the reverse must be true for *S*_*iK*_ → *S*_*iL*_. We then collect all orbital sets that fail this mapping condition (*m*_*LK*_(*S*_*iL*_) = {}) for some structure combination because they contain an inconsistent orbital mapping. The orbitals in these sets are assigned to the second set of nonmatchable orbitals .

All remaining orbital sets are unambiguously identifiable in all structures according to the mapping conditions. They are mapped through the final bijective map
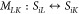
6

The map *M*_*LK*_ is bijective and maps orbital sets of structures *L* and *K* that are of the same size. Furthermore, we define a map *A* between the sets of nonmatchable orbitals .
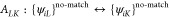
7

Because the total numbers of orbitals in each set  are always the same, the sets  will always have the same size. They contain the orbitals that change during a reaction, e.g., orbitals involved in bond-breaking/bond-formation processes. The maps *M*_*LK*_ and *A*_*LK*_ combined provide bijective maps between orbital sets containing every orbital of  of every structure.

The mapping algorithm requires orbitals that are transferable between structures. Otherwise, the sets of nonmatchable orbitals  become large, and the mapping is useless. To maximize the transferability of the orbitals, they are localized before the mapping. We found that the intrinsic bond orbital scheme^36,37^ provides highly transferable orbitals for this purpose.

We first localize only the orbitals of one structure and then align all other orbital sets to the already localized orbitals by minimizing the difference in orbital populations to these template orbitals.^38^ Then, all other orbital sets are localized. This increases the chance that all orbital localization procedures converge to similar orbitals. Note that the specific choice of the template orbital set to which all other orbital sets are aligned will influence the final result of the orbital localization. However, it was shown previously^38^ that the choice in template orbitals is unlikely to have an effect on the transferability of the orbitals between structures which is the key quantity for the orbital mapping.

The only input parameter for the mapping procedure is the orbital similarity threshold τ [see eq 1, and τ = τ_kin_ = τ_loc_]. However, if we are only interested in a qualitative mapping of the orbitals along the reaction coordinate, we can eliminate τ by requiring that the set  becomes as small as possible, while it remains reasonable for comparing orbital populations (in this work τ ≤ 0.5).
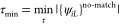
8

Note that  does not necessarily decrease with increasing τ because an increasingly loose mapping through eq 1 leads to increasingly inconsistent orbital maps *m*_*LK*_.

We now combine the mapping procedure with the automated active space selection procedure implemented in autoCAS.^18,34^autoCAS selects active orbital spaces based on single-orbital entropies calculated by low-cost DMRG configuration interaction (CI) calculations for the entire valence orbital space. These DMRG-CI calculations are carried out by our DMRG program QCMaquis.^39^

To fully automatize the approach, we interfaced the quantum chemistry software Serenity(40−42) to autoCAS. Serenity implements the orbital-mapping, orbital-alignment, and orbital-localization procedures discussed above.

The complete approach for a set of structures along a reaction coordinate consists of the following steps:

(1) Calculate the HF orbitals for all structures.
(2) Localize and align the occupied and virtual valence orbitals.
(3) Construct the orbital set maps *A*_*LK*_ and *M*_*LK*_.
(4) Select active orbital sets with autoCAS for all structures (or any subset of structures if it is known that more structures will add no additional orbitals to the active orbital set).
(5) Construct the active orbital sets for each structure as the union of all sets, which are mapped to a set containing at least one orbital selected by autoCAS.
(6) Converge the final active space calculations.

To demonstrate that active orbital spaces can quickly become inconsistent if the electronic character changes significantly along a reaction coordinate, we study the case of homolytic bond dissociation. For stable intermediates such as reactants, a single determinant often describes the electronic structure well. However, a multiconfigurational description of the wave function is required upon dissociation. As an example, we investigate the potential energy curve of the homolytic bond dissociation of 1-pentene, as shown in Figure 4. The structures of the potential energy curve are obtained through curve optimization of the minimum energy path between the bonded and fully dissociated fragments.^43^ This was carried out with the program Readuct(44) applying the PBE exchange–correlation functional^45^ with spin-unrestricted orbital optimization, Grimme’s D3 dispersion correction,^46^ Becke–Johnson damping,^47^ and the cc-pVDZ basis set^48^ in Turbomole(49) raw data calculations. We selected 22 structures along this potential energy curve, which are provided in the Supporting Information. For these structures, we calculated the localized and aligned spin-restricted HF orbitals in the cc-pVDZ basis set.^48^ We selected the active orbital space with autoCAS only for the separated product to reduce the number of DMRG-CI calculations. The separated product showed the strongest multireference character, and its active orbital space already contains all orbitals that are needed in the active orbital space for all structures along the reaction coordinate. Furthermore, we chose a low bond dimension of 250 and a low number of (back-and-forth) sweeps of only 5 for the DMRG-CI calculations, which limit the computational overhead of the active space selection but provide a qualitatively correct description of the single-orbital entropies (illustrated in Table 1 for the highest eight single orbital entropies). The single-orbital entropies are converged up to the fourth digit compared to DMRG-CI calculations with increased bond dimension (up to 1200) and increased number of DMRG sweeps (up to 10). We then transferred the active orbital space to all other structures. The selection thresholds for occupied and virtual orbitals were optimized according to eq 8 (occupied: τ_min_ = 0.5, virtual: τ_min_ = 0.5). To demonstrate that the orbital mapping procedure is insensitive to the explicit choice of τ, we provide a plot of  with respect to τ in Figure 2. The size of  is stable at 1 for the virtual and occupied orbitals before it increases for values of small τ < 0.435.

**Table 1:** Eight Highest Single-Orbital Entropies Calculated with DMRG-CI for Increasing Bond Dimension (*m*) and Number of DMRG Sweeps (*w*) for the Product of the Homolytic Carbon–Carbon Bond Dissociation of 1-Pentene (Both Parameters Are Denoted as *m*/*w* in the 2nd to 5th Column)

orbital index	250/5	800/5	800/10	1200/5
5	1.06029	1.06025	1.06025	1.06025
19	1.01578	1.01574	1.01574	1.01574
23	0.99425	0.99435	0.99435	0.99435
32	0.33259	0.33270	0.33270	0.33271
22	0.20515	0.20517	0.20517	0.20517
34	0.11351	0.11355	0.11355	0.11355
28	0.11333	0.11337	0.11337	0.11338
21	0.10647	0.10652	0.10652	0.10652

**Figure 2:**
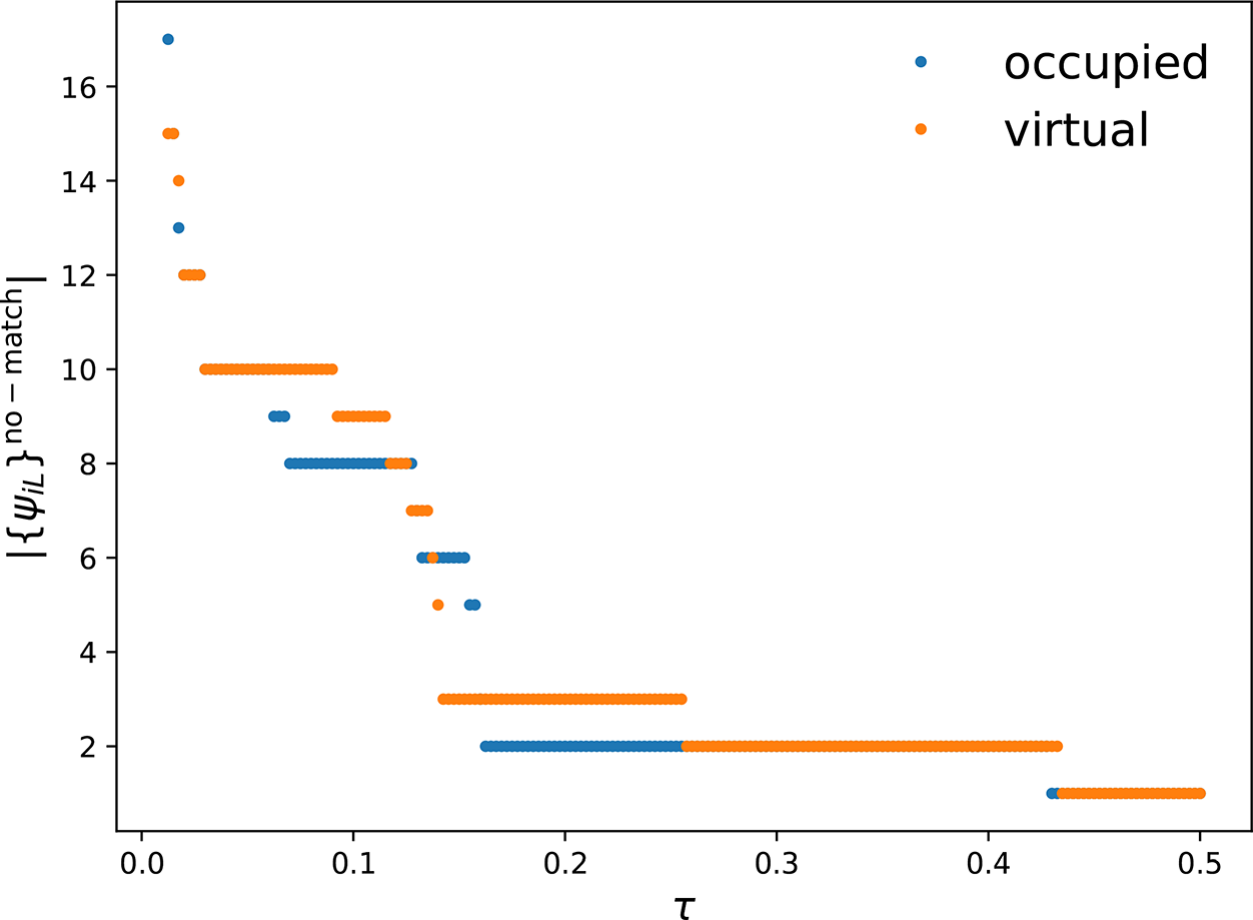
Number of orbitals in the sets  for virtual (orange) and occupied (blue) orbitals as a function of . Lowering leads to a stepwise increase of the size of  from the right to the left.

The orbital mapping of the localized virtual and occupied valence orbitals is shown in Figure 3 for a selection of orbitals. In this example, every orbital set from the maps *A*_*LK*_ and *M*_*LK*_ has only one element; i.e., the maps provide a bijection between orbitals. The only two nonmatchable orbitals are the bonding and antibonding carbon–carbon σ-bond orbitals of the bond broken upon reaction. The localized orbitals are very compact and represent the expected nodal structure of bonding and antibonding σ- and π-orbitals.

**Figure 3:**
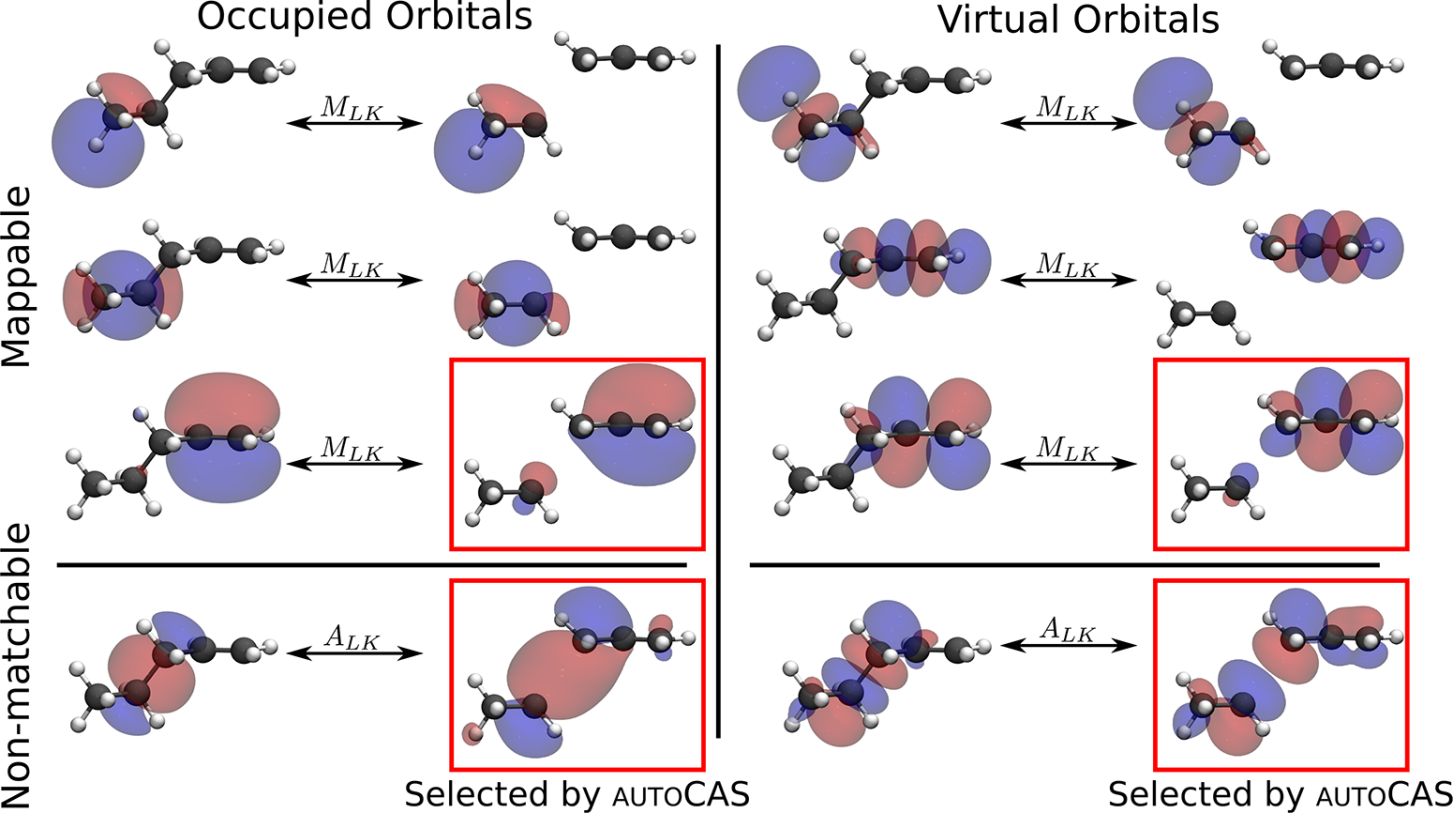
Orbital set mapping through *M*_*LK*_ and *A*_*LK*_ for valence virtual and occupied orbitals of 1-pentene in its equilibrium and almost dissociated structures. Orbital isosurfaces are shown for a value of ±0.025 au. The red boxes highlight the orbitals selected by autoCAS. The orbitals for the fourth (equilibrium) and 12th structures are shown (the structure indices increase with increasing carbon–carbon internuclear distance). All structures are provided in the Supporting Information.

The orbitals selected by autoCAS are highlighted in Figure 3 by red boxes. autoCAS identified the bonding and antibonding σ-carbon–carbon bond orbitals and the bonding and antibonding π-orbitals as statically correlated. These orbitals are then included in the active orbital space for a CASSCF calculation with second-order perturbation theory^50,51^ (CASPT2, without any IPEA shift^52^). Note that for active spaces with more than about 20 orbitals, which are beyond the capabilities of traditional CASSCF, DMRG-SCF plus subsequent perturbation theory can be used in QCMaquis.^53,54^ The potential energy curve calculated with this consistent active orbital space and CASPT2 (without IPEA shift) is denoted as CASPT2(4,4) and shown in Figure 4. Furthermore, we show the potential energy curves calculated with CASPT2 and other choices in the active orbital space. We selected the active orbital space with autoCAS from the canonical HF orbitals without ensuring that these spaces are consistent between structures. We denote the CASPT2 result calculated with this active space choice as CASPT2(inconsistent). In addition, we selected active orbital spaces as only the highest occupied (HOMO) and lowest unoccupied (LUMO) canonical HF orbital, leading to a CAS(2,2) [denoted as CASPT2(2,2)]. In addition, we show below the potential energy profiles calculated with coupled cluster theory with singles, and doubles excitations and perturbatively treated triples excitations [CCSD(T)]^55^ as implemented in Turbomole.^49^ The CCSD(T) energies were calculated with spin-restricted orbitals.

**Figure 4:**
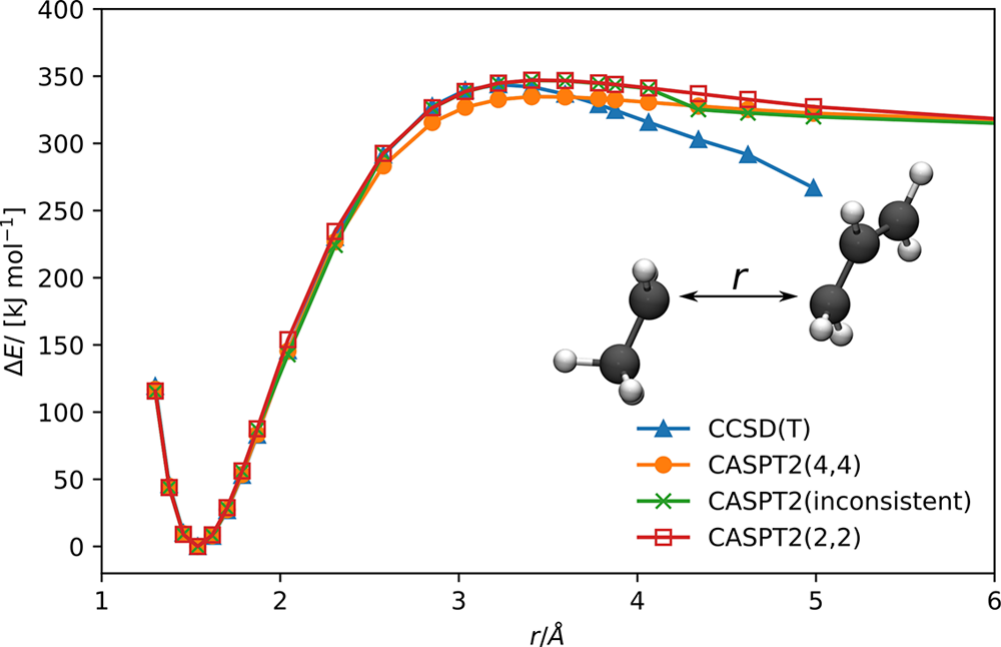
Potential energy profile for the homolytic carbon–carbon bond dissociation in 1-pentene. The energy of the minimum structure is chosen as the zero energy reference point. For CASPT2(4,4), the active space was selected for the dissociated fragments and then transferred with the orbital mapping to all structures along the reaction coordinate. For CASPT2(inconsistent), the active space was selected with autoCAS for each structure along the reaction coordinated without ensuring its consistency. For CASPT2(2,2), only the HOMO and the LUMO are included in the active space.

The CASPT2 potential energy curves agree with the CCSD(T) potential energy curve for short internuclear carbon–carbon distances (*r* < 3.4 Å), independent of the choice in the active space. For large internuclear distances (*r* > 3.4 Å), the CASPT2 curves and the CCSD(T) curve show significant differences. All CASPT2 curves converge to a similar, and constant relative energy, which are 314.8 kJ mol^–1^ for CASPT2(4,4), 312.3 kJ mol^–1^ for CASPT2(inconsistent), and 313.3 kJ mol^–1^ for CASPT2(2,2). By contrast, the relative energy for CCSD(T) nonphysical decreases. In fact, the CCSD(T) calculation failed for the fragments with the largest carbon–carbon internuclear distance.

The CASPT2 curves show significant differences for internuclear distances between 2.5 and 5.0 Å. For these distances, CASPT2(2,2) and CASPT2(inconsistent) give very close results with slightly higher relative energies than CASPT2(4,4). The relative energies for CASPT2(2,2) and CASPT2(inconsistent) are higher because of the smaller active space. The active spaces for the structures between 3.7 and 4.1 Å contain in the cases of CASPT2(2,2) and CASPT2(inconsistent) only the HOMO and the LUMO. However, for distances *r* > 4.3 Å, the bonding and antibonding π-orbitals are selected for the active space by autoCAS in addition to the HOMO and the LUMO. Therefore, the CASPT2(inconsistent) potential energy curve shows a sharp kink corresponding to this transition from a CAS(2,2) to a CAS(4,4). The active orbital spaces for CASPT2(inconsistent) show this inconsistency because autoCAS only ensures that the active space is suitable for the given structure and does not take any other structures on the potential energy curve into account. By contrast, the CASPT2(4,4) potential energy curve is smooth for all internuclear distances investigated here because our algorithm ensures that the active orbital space is consistent between all structures.

To illustrate our approach for a second reaction coordinate, we investigated the rotation of the CH_2_ group around the carbon–carbon double bond in 1-pentene by varying the H–C–C–H dihedral angle ϕ between 0° and 180°, as illustrated in Figure 5a. The active space selection was performed only for the angles ϕ = 0° and 90° from the aligned and localized HF orbitals calculated in the cc-pVDZ basis set.^48^ The combined active orbital spaces were then transferred to all other structures through the orbital mapping procedure (occupied: τ_min_ = 0.5, virtual: τ_min_ = 0.5). The bonding and antibonding π-orbitals were selected by autoCAS for the active orbital space of both structures. For these orbitals no match could be realized during the mapping procedure, leading to their assignment to the orbital sets . The isosurface plots of the π-orbitals are shown in Figure 5b. The energy profile of the rotation of the CH_2_ group is shown in Figure 5, calculated with CASPT2 for this automatically selected and transferred active orbital space.

**Figure 5:**
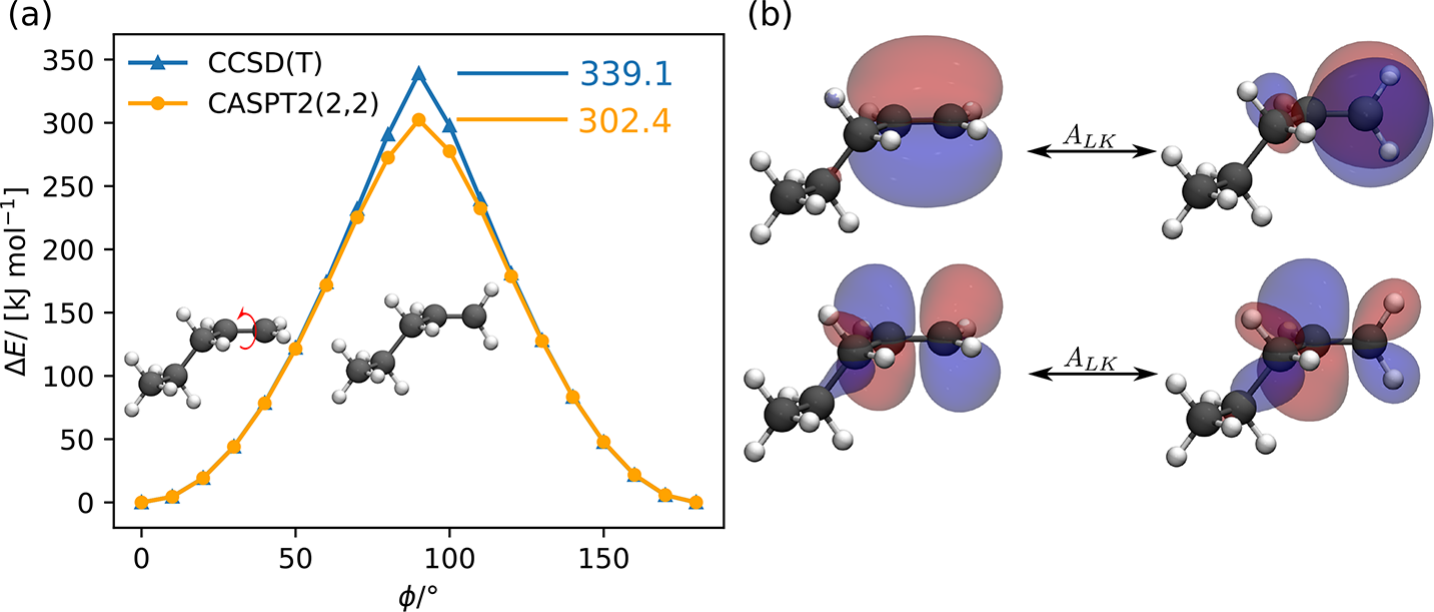
(a) Energy profile of the CH_2_ group rotation around the carbon–carbon double bond of 1-pentene. The CAS(2,2) for CASPT2 was constructed by automatically selecting an active orbital space with autoCAS for the rotation angles ϕ = 0° and ϕ = 90° and transferring the combined active space to all other structures. (b) Orbital map of the orbitals selected for the active orbital space by autoCAS and CH_2_ rotation around the carbon–carbon double bond. Orbital isosurfaces are shown for a value of ±0.025 au.

Furthermore, we show the energy profile for this rotation calculated with CCSD(T). CCSD(T) predicts the barrier height for the rotation to be 36.7 kJ mol^–1^ higher than CASPT2. The point of the maximum energy corresponds to the dihedral angle of ϕ = 90°. In this case, the electronic structure shows a significant multireference character, as illustrated by the high value of 0.1930 for the *D*1 diagnostic^56^ calculated from the CCSD amplitudes (values of *D*1 < 0.05 indicate a single reference character of the wave function^56^), indicating that CCSD(T) is not a suitable wave function model to describe the electronic structure correctly for ϕ = 90° and that CASPT2 should provide a more reliable description. For angles 0°–70° and 110°–180°, the CASPT2 and CCSD(T) energy profiles agree well. Furthermore, the CASPT2 energy profile is smooth, showing that transferring active orbital spaces between structures with our mapping approach is reliable.

In this work, we demonstrated how occupied, and virtual orbitals can be matched between structures along a path on the Born–Oppenheimer hypersurface (such as a reaction coordinate). Our approach provides a rigorous protocol for constructing consistent active orbital spaces for CAS calculations for chemical reactions. The protocol requires no input arguments, is fully automatized, and is integrated with the automatic active orbital selection approach implemented in autoCAS.

In future work, we will investigate the effect of tailoring the active space selection directly to the reaction by choosing the comparison threshold τ explicitly similar to the original embedding region selection presented in ref (24). Moreover, we will consider the straightforward extension to electronically excited states.

## Supporting Information Available

Coordinate files of the potential energy curve and a python script that carries out all calculations described in this work. (ZIP)
